# Dexmedetomidine-Induced Aortic Contraction Involves Transactivation of the Epidermal Growth Factor Receptor in Rats

**DOI:** 10.3390/ijms23084320

**Published:** 2022-04-13

**Authors:** Soo Hee Lee, Seong-Chun Kwon, Seong-Ho Ok, Seung Hyun Ahn, Sung Il Bae, Ji-Yoon Kim, Yeran Hwang, Kyeong-Eon Park, Mingu Kim, Ju-Tae Sohn

**Affiliations:** 1 Department of Anesthesiology and Pain Medicine, Gyeongsang National University Changwon Hospital 11, Samjeongja-ro, Seongsan-gu, Changwon-si 51472, Gyeongsangnam-do, Korea; lishiuji@naver.com (S.H.L.); mdoksh@naver.com (S.-H.O.); 2 Department of Anesthesiology and Pain Medicine, Gyeongsang National University College of Medicine, 15 Jinju-daero 816 beon-gil, Jinju-si 52727, Gyeongsangnam-do, Korea; 3 Institute of Health Sciences, Gyeongsang National University, Jinju-si 52727, Gyeongsangnam-do, Korea; 4 Department of Physiology, Institute of Clinical and Translational Research, Catholic Kwandong University, College of Medicine, Gangneung 25601, Korea; skwon2028@cku.ac.kr; 5 Department of Anesthesiology and Pain Medicine, Gyeongsang National University Hospital, 15 Jinju-daero 816 beon-gil, Jinju-si 52727, Gyeongsangnam-do, Korea; ash3832@gmail.com (S.H.A.); snugsoul@naver.com (S.I.B.); avadore33@naver.com (J.-Y.K.); babi9876@naver.com (Y.H.); kep1574@naver.com (K.-E.P.); mingu1874@naver.com (M.K.); 6 Department of Anesthesiology and Pain Medicine, Gyeongsang National University College of Medicine, Gyeongsang National University Hospital, 15 Jinju-daero 816 beon-gil, Jinju-si 52727, Gyeongsangnam-do, Korea

**Keywords:** dexmedetomidine, contraction, epidermal growth factor receptor, transactivation, alpha-2 adrenoceptor

## Abstract

In this study, we examined whether aortic contraction, induced by the alpha-2 adrenoceptor agonist dexmedetomidine, is involved in the transactivation of the epidermal growth factor receptor (EGFR) in isolated endothelium-denuded rat aortas. Additionally, we aimed to elucidate the associated underlying cellular mechanisms. The effects of the alpha-2 adrenoceptor inhibitor rauwolscine, EGFR tyrosine kinase inhibitor AG1478, Src kinase inhibitors PP1 and PP2, and matrix metalloproteinase inhibitor GM6001 on EGFR tyrosine phosphorylation and c-Jun NH_2_-terminal kinase (JNK) phosphorylation induced by dexmedetomidine in rat aortic smooth muscles were examined. In addition, the effects of these inhibitors on dexmedetomidine-induced contraction in isolated endothelium-denuded rat aorta were examined. Dexmedetomidine-induced contraction was inhibited by the alpha-1 adrenoceptor inhibitor prazosin, rauwolscine, AG1478, PP1, PP2, and GM6001 alone or by a combined treatment with prazosin and AG1478. AG1478 (3 × 10^−6^ M) inhibited dexmedetomidine-induced contraction in isolated endothelium-denuded rat aortas pretreated with rauwolscine. Dexmedetomidine-induced EGFR tyrosine and JNK phosphorylation were inhibited by rauwolscine, PP1, PP2, GM6001, and AG1478. Furthermore, dexmedetomidine-induced JNK phosphorylation reduced upon EGFR siRNA treatment. Therefore, these results suggested that the transactivation of EGFR associated with dexmedetomidine-induced contraction, mediated by the alpha-2 adrenoceptor, Src kinase, and matrix metalloproteinase, caused JNK phosphorylation and increased calcium levels.

## 1. Introduction

The alpha-2 adrenoceptor agonist dexmedetomidine is used as a sedative in operating rooms and intensive care units [1,2]. The intravenous administration of a large dose of dexmedetomidine (1 or 2 μg/kg) produces a biphasic response, characterized by a transient increase and then a decrease in blood pressure [3]. The dexmedetomidine-induced transient increase in blood pressure is associated with alpha-2 adrenoceptor stimulation of the vascular smooth muscle [4]. A high plasma concentration of dexmedetomidine (8.4 and 14.7 ng/mL) in humans increases the mean arterial blood pressure and systemic vascular resistance while causing decreased cardiac output, which seems to be associated with the vasoconstriction that is observed when this drug is administered. [4,5]. Accordingly, when a high dose of dexmedetomidine (4 μg/kg/h) is used for sedation in pediatric patients, hypertension or bradycardia is observed, which seems to be due to the vasoconstriction induced by alpha-2B adrenoceptor stimulation of the vascular smooth muscle [6,7,8].

The activation of epidermal growth factor receptor (EGFR) is involved in blood pressure regulation and endothelial dysfunction [9]. EGFR transactivation, which is induced without direct interaction with G-protein-coupled agonists, is associated with signaling pathway proteins, such as Src kinase, reactive oxygen species, protein kinase C, calcium, matrix metalloproteinase (MMP), and extracellular-signal-regulated protein kinase or c-Jun NH_2_-terminal kinase (JNK) [9]. Epidermal growth factors induce the contraction of aortic strips and increase cytoplasmic calcium concentration [10]. In addition, dexmedetomidine-induced contraction is mediated by JNK in isolated rat aortas and is dependent on the calcium influx from the extracellular space that enters via voltage-gated calcium channels [11,12]. Mitogen-activated protein kinase phosphorylation, stimulated by the alpha-2B adrenoceptor in renal tubular cell lines, is mediated by arachidonic acid, MMP, and the transactivation of EGFR [13]. Dexmedetomidine-induced EGFR transactivation in astrocytes is mediated by MMP and Src kinase [14]. However, the role of EGFR in the muscle contraction that is induced by dexmedetomidine remains unknown. Therefore, the goal of this study was to examine whether dexmedetomidine-induced contraction involves the transactivation of EGFR in an isolated rat aorta and to elucidate the associated cellular signaling pathways. Based on previous reports, we tested the novel hypothesis that dexmedetomidine-induced EGFR transactivation is associated with the pathways involving Src kinase, MMP, EGFR, and JNK.

## 2. Results

**Figure 1:**
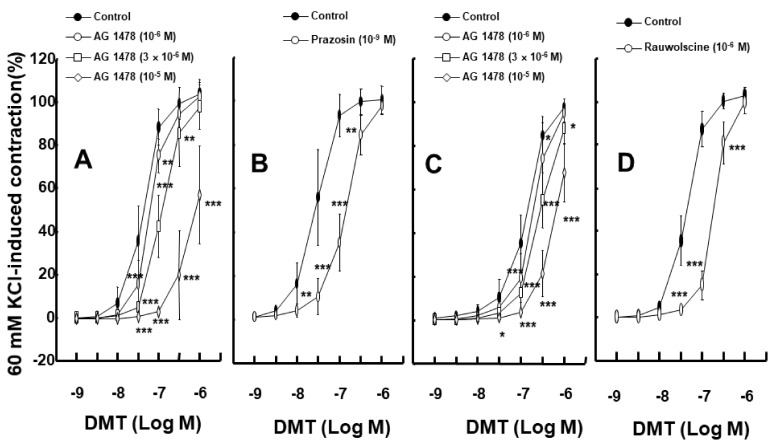
(**A**,**B**): Effects of AG1478 ((**A**) N = 8, 6, 5, and 6 for control, 10^−6^, 3 **×** 10^−6^, and 10^−5^ M, respectively) and prazosin ((**B**) N = 5) on dexmedetomidine (DMT)-induced contraction in isolated endothelium-denuded rat aortas. (**C**): Effect of AG1478 (N = 5) on DMT-induced contraction in isolated endothelium-denuded rat aortas pretreated with prazosin (10^−9^ M). (**D**): Effect of rauwolscine (10^−6^ M, N = 5) on DMT-induced contraction in isolated endothelium-denuded rat aortas. Data are shown as mean ± SD and are expressed as a percentage of isotonic 60 mM KCl-induced contraction. N indicates the number of rats. Control indicates no treatment. * *p* < 0.05, ** *p* < 0.01, and *** *p* < 0.001 versus control.

**Figure 2:**
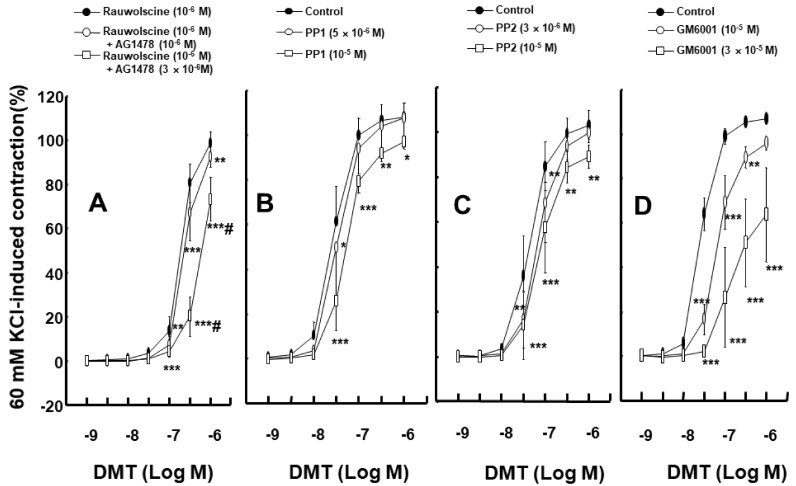
(**A**): Effect of AG1478 (N = 7, 6, and 5 for rauwolscine alone, rauwolscine plus 10^−6^ M AG1478, and rauwolscine plus 3 × 10^−6^ M AG1478, respectively) on dexmedetomidine (DMT)-induced contraction in isolated endothelium-denuded rat aortas pretreated with 10^−6^ M rauwolscine. Data are shown as mean ± SD and are expressed as a percentage of isotonic 60 mM KCl-induced contraction. N indicates the number of rats. ** *p* < 0.01 and *** *p* < 0.001 versus rauwolscine alone. # *p* <0.001 versus 10^−6^ M rauwolscine plus 10^−6^ M AG1478. (**B**–**D**): Effects of PP1 ((**B**) N = 6, 5, and 6 for control, 5 × 10^−6^, and 10^−5^ M PP1, respectively), PP2 ((**C**) N = 6, 5, and 6 for control, 3 × 10^−6^, and 10^−5^ M PP2, respectively), and GM6001 ((**D**) N = 5, 5, and 6 for control, 10^−5^ and 3 × 10^−5^ M GM6001, respectively) on DMT-induced contraction in isolated endothelium-denuded rat aortas. Data are shown as mean ± SD and are expressed as a percentage of isotonic 60 mM KCl-induced contraction. N indicates the number of rats. Control indicates no treatment. * *p* < 0.05, ** *p* < 0.01, and *** *p* < 0.001 versus control.

To test whether dexmedetomidine-induced EGFR transactivation is associated with Src kinase, MMP, and EGFR, we used inhibitors to determine if there were any effects. Dexmedetomidine-induced contraction in an endothelium-denuded rat aorta was attenuated by the EGFR tyrosine kinase inhibitor, AG1478, at 10^−6^ to 10^−5^ M concentrations (Figure 1A; *p* < 0.01 versus control at 3 × 10^−8^ and 10^−7^ M). Dexmedetomidine-induced contraction was also inhibited by the 10^−9^ M alpha-1 adrenoceptor inhibitor prazosin (Figure 1B; *p* < 0.01 versus control at 10^−8^ to 3 × 10^−7^ M). In addition, dexmedetomidine-induced contraction in an endothelium-denuded aorta, which was pretreated with prazosin at 10^−9^ M concentration, was inhibited by AG1478 when using concentrations of 10^−6^ to 10^−5^ M (Figure 1C; *p* < 0.05 versus control at 10^−7^ and 3 × 10^−7^ M). The alpha-2 adrenoceptor inhibitor rauwolscine (10^−6^ M) inhibited dexmedetomidine-induced contraction (Figure 1D; *p* < 0.001 versus control at 3 × 10^−8^ to 3 × 10^−7^ M). AG1478 (10^−6^ and 3 × 10^−6^ M) inhibited dexmedetomidine-induced contraction in endothelium-denuded rat aortas pretreated with rauwolscine (10^−6^ M) (Figure 2A; *p* < 0.01, versus rauwolscine alone at 10^−7^ to 10^−6^ M). Additionally, AG1478 at 3 × 10^−6^ M had a stronger inhibitory effect on dexmedetomidine-induced contraction than AG1478 at 10^−6^ M in endothelium-denuded rat aortas pretreated with rauwolscine (10^−6^ M) (Figure 2A; *p* < 0.001 at 3 × 10^−7^ and 10^−6^ M). Dexmedetomidine-induced contraction of an endothelium-denuded rat aorta was inhibited by the Src kinase inhibitor PP1 at a concentration of 5 × 10^−6^ and 10^−5^ M (Figure 2B; *p* < 0.05 versus control at 3 × 10^−8^ M). In addition, similar inhibitory results were found by using the Src kinase inhibitor PP2 at concentrations of 3 × 10^−6^ and 10^−5^ M (Figure 2C; *p* < 0.01, versus control at 3 × 10^−8^ and 10^−7^ M), as well as using the MMP inhibitor GM6001 at concentrations of 10^−5^ and 3 × 10^−5^ M (Figure 2D; *p* <0.01 versus control at 3 × 10^−8^ to 3 ×10^−7^ M). However, dexmedetomidine-induced contraction was not altered by dimethyl sulfoxide (DMSO, 0.1%) or ethanol (0.1%), which was used for the dissolution of AG1478, PP1, PP2, and GM6001 (Supplementary Figure S1). 

**Figure 3:**
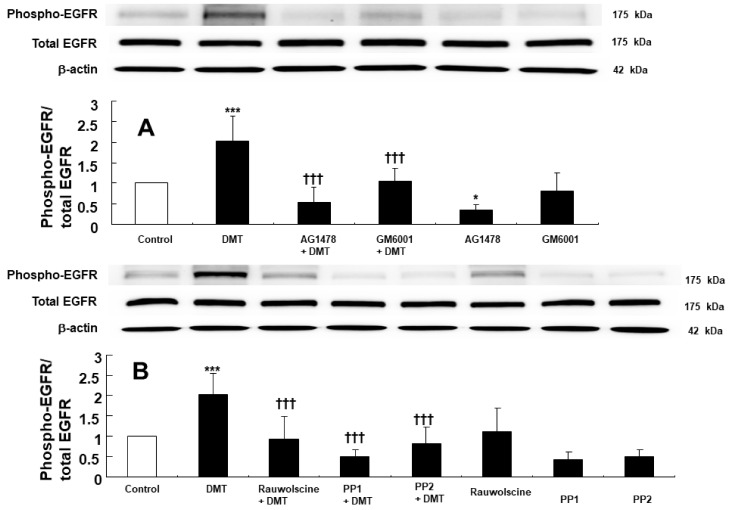
(**A**): Effects of AG1478 (10^−5^ M) and GM6001 (3 **×** 10^−5^ M) on dexmedetomidine (DMT, 10^−6^ M)-induced epidermal growth factor receptor (EGFR) phosphorylation (Tyr1068) in rat aortic vascular smooth muscle. Data (N = 6) are shown as mean ± SD. N indicates the number of independent experiments. Control indicates no treatment. * *p* < 0.05 and *** *p* < 0.001 versus control. ††† *p* < 0.01 versus DMT alone. (B): Effects of rauwolscine (10^−6^ M), PP1 (10^−5^ M), and PP2 (10^−5^ M) on the DMT-induced EGFR phosphorylation (Tyr1068) in rat aortic vascular smooth muscle. Data (N = 6) are shown as mean ± SD. N indicates the number of independent experiments. Control indicates no treatment. *** *p* < 0.001 versus control. ††† *p* < 0.01 versus DMT alone.

**Figure 4:**
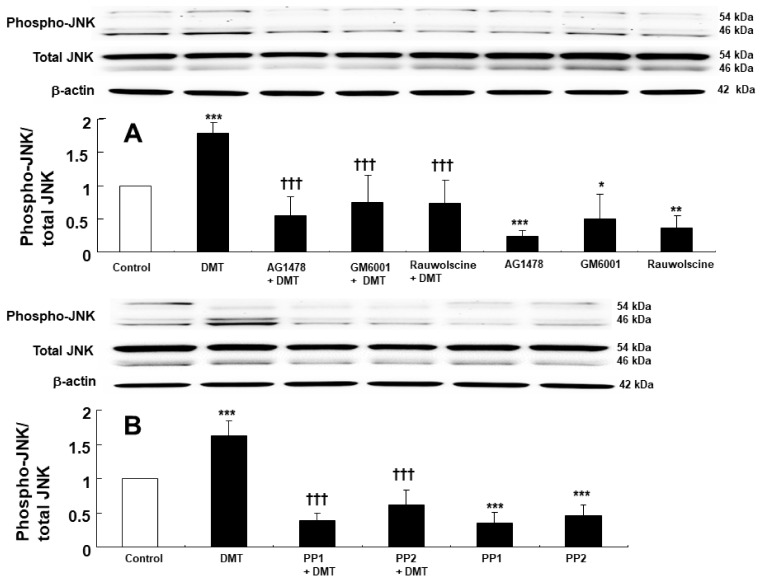
(**A**): Effects of AG1478 (10^−5^ M), GM6001 (3 × 10^−5^ M), and rauwolscine (10^−6^ M) on the c-Jun NH_2_-terminal kinase (JNK) phosphorylation induced by dexmedetomidine (DMT, 10^−6^ M) in rat aortic vascular smooth muscle. Data (N = 5) are shown as mean ± SD. N indicates the number of independent experiments. * *p* < 0.05, ** *p* < 0.01, and *** *p* < 0.001 versus control. Control indicates no treatment. ††† *p* < 0.001 versus DMT alone. (**B**): Effects of PP1 (10^−5^ M) and PP2 (10^−5^ M) on the JNK phosphorylation induced by DMT in rat aortic vascular smooth muscle. Data (N = 5) are shown as mean ± SD. N indicates the number of independent experiments. Control indicates no treatment. *** *p* < 0.001 versus control. ††† *p* < 0.001 versus DMT alone.

**Figure 5:**
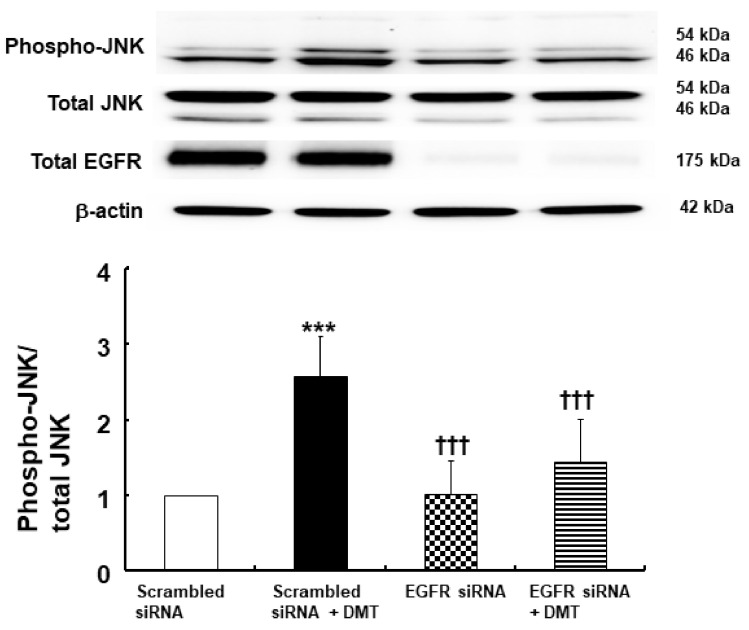
Effect of dexmedetomidine (DMT, 10^−6^ M) on c-Jun NH_2_-terminal kinase (JNK) phosphorylation in rat aortic vascular smooth muscle cells transfected with scrambled siRNA and epidermal growth factor receptor (EGFR) siRNA. Data (N = 4) are shown as mean ± SD. N indicates the number of independent experiments. *** *p* < 0.001 versus scrambled siRNA. ††† *p* < 0.001 versus scrambled siRNA + DMT.

EGFR phosphorylation (at Tyr1068) in rat aortic smooth muscle was induced by 10^−6^ M dexmedetomidine (Figure 3A,B; *p* < 0.001, versus control). However, dexmedetomidine-induced EGFR phosphorylation (Tyr1068) was inhibited by the EGFR tyrosine kinase inhibitor AG1478 at a concentration of 10^−5^ M and was similarly inhibited using the MMP inhibitor GM6001 at a concentration of 3 × 10^−5^ M (Figure 3A; *p* < 0.001, versus dexmedetomidine alone). In addition, dexmedetomidine-induced EGFR phosphorylation (Tyr1068) was inhibited by the alpha-2 adrenoceptor inhibitor rauwolscine at a 10^−6^ M concentration and also using the Src kinase inhibitors, PP1 at 10^−5^ M and PP2 at 10^−5^ M (Figure 3B; *p* < 0.001 versus dexmedetomidine alone). JNK phosphorylation in rat aortic smooth muscle was induced by 10^−6^ M dexmedetomidine treatment (Figure 4A,B; *p* < 0.001, versus control). Dexmedetomidine-induced JNK phosphorylation was inhibited by the addition of 10^−5^ M of the EGFR tyrosine kinase inhibitor AG1478, 3 × 10^−5^ M of the MMP inhibitor GM6001, and 10^−6^ M of the alpha-2 adrenoceptor inhibitor rauwolscine (Figure 4A; *p* < 0.001 versus dexmedetomidine). Dexmedetomidine-induced JNK phosphorylation in rat aortic vascular smooth muscle was also inhibited by the Src kinase inhibitors, PP1 at a 10^−5^ M concentration and PP2 at a 10^−5^ M concentration (Figure 4B; *p* < 0.001 versus dexmedetomidine alone). Furthermore, JNK phosphorylation of rat aortic vascular smooth muscle cells transfected with scrambled small interfering RNA (siRNA) was induced by dexmedetomidine (10^−6^ M) (Figure 5; *p* <0.001 versus scrambled siRNA). However, when rat aortic vascular smooth muscle cells transfected with EGFR siRNA were treated with dexmedetomidine, JNK phosphorylation was lower than with scrambled siRNA (Figure 5; *p* < 0.001). As a control, we ensured that total EGFR expression was reduced in rat aortic vascular smooth muscle cells transfected with EGFR siRNA (Figure 5).

**Figure 6:**
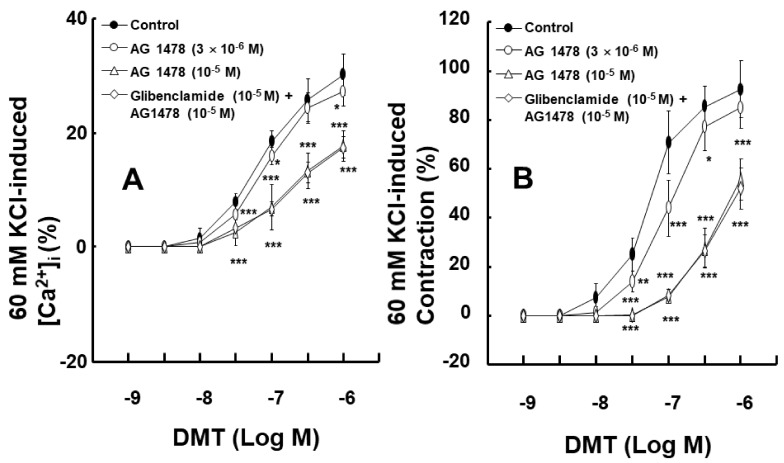
Effects of AG1478 alone and the combined treatment with glibenclamide and AG1478 on the intracellular calcium level ([Ca^2+^]_i_) (**A**) and contraction (**B**) induced by dexmedetomidine (DMT) in endothelium-denuded aortic strips loaded with Fura-2. The [Ca^2+^]_i_ and contraction induced by DMT are expressed as a percentage of [Ca^2+^]_i_ and contraction induced by 60 mM KCl, respectively. Data (N = 5) are shown as mean ± SD. N indicates the number of independent experiments. Control indicates no treatment. * *p* < 0.05, ** *p* < 0.01, and *** *p* < 0.001 versus control.

**Figure 7:**
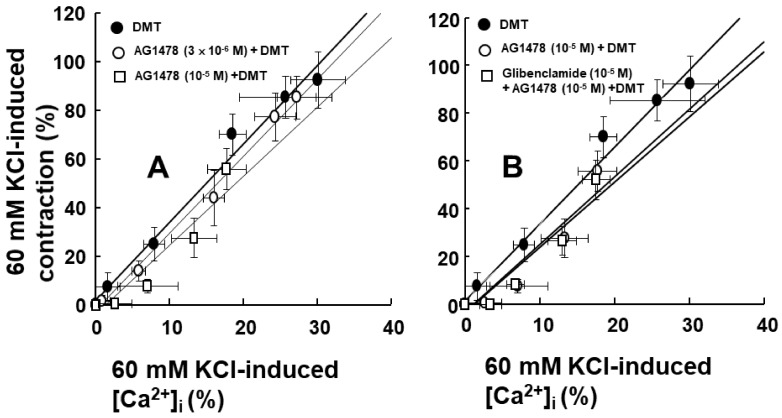
Intracellular calcium level ([Ca^2+^]_i_)–tension relationship induced by dexmedetomidine (DMT; 10^−9^ to 10^−6^ M) in the presence or absence of AG1478 alone (**A**) or with glibenclamide plus AG1478 (**B**). [Ca^2+^]_i_ and contraction induced by DMT are expressed as a percentage of [Ca^2+^]_i_ and contraction induced by 60 mM KCl, respectively. Each point shows the mean of five experiments, and SD is expressed as the vertical and horizontal bars.

To examine the impact of EGFR on intracellular calcium levels [Ca^2+^]_i_ and the contraction induced by dexmedetomidine, [Ca^2+^]_i_ and contraction were induced by dexmedetomidine in Fura-2-loaded aortic strips. These processes were inhibited by the EGFR tyrosine kinase inhibitor, AG1478, at concentrations of 3 × 10^−6^ and 10^−5^ M ([Ca^2+^]_i_: Figure 6A, *p* < 0.05 versus control at 10^−7^ and 10^−6^ M; contraction: Figure 6B, *p* < 0.05 versus control at 3 × 10^−8^ to 3 × 10^−7^ M). However, [Ca^2+^]_i_ and contraction were not affected by 10^−5^ M of the adenosine triphosphate (ATP)-sensitive potassium channel inhibitor glibenclamide in the presence of 10^−5^ M AG1478 (Figure 6A,B). The slope of simultaneous [Ca^2+^]_i_ and tension induced by dexmedetomidine was not significantly altered by AG1478 at concentrations of 3 × 10^−6^ or 10^−5^ M (Figure 7A; dexmedetomidine alone = 3.17 ± 0.34; 3 × 10^−6^ M AG1478 plus dexmedetomidine = 3.16 ± 0.49; 10^−5^ M AG1478 plus dexmedetomidine = 2.80 ± 0.53). In addition, the slope of simultaneous [Ca^2+^]_i_ and tension induced by dexmedetomidine in the presence of AG1478 at 10^−5^ M was not affected by 10^−5^ M glibenclamide (Figure 7B; 10^−5^ M AG1478 plus dexmedetomidine = 2.80 ± 0.53 versus 10^−5^ M glibenclamide plus 10^−5^ M AG1478 plus dexmedetomidine = 2.69 ± 0.46), suggesting that the AG1478-induced inhibition of [Ca^2+^]_i_ and tension, induced by dexmedetomidine, is not mediated by ATP-sensitive potassium channels.

## 3. Discussion

This is the first study to suggest that dexmedetomidine-induced contraction of aortic smooth muscle occurs via the transactivation of EGFR, which is activated by a pathway involving the alpha-2 adrenoceptor, Src kinase, and MMP, and then induces JNK phosphorylation. The major findings of this study are as follows: (1) AG1478, PP1, PP2, and GM6001 attenuated dexmedetomidine-induced contraction; (2) AG1478 inhibited dexmedetomidine-induced contraction in aortas pretreated with the alpha-2 adrenoceptor inhibitor rauwolscine; (3) rauwolscine, AG1478, PP1, PP2, and GM6001 inhibited EGFR and JNK phosphorylation induced by dexmedetomidine; (4) EGFR siRNA inhibited dexmedetomidine-induced JNK phosphorylation; and (5) AG1478 attenuated [Ca^2+^]_i_ increase by dexmedetomidine.

Angiotensin II induces EGFR transactivation, which is mediated by a pathway involving the angiotensin II receptor and the shedding of the metalloproteinase-dependent, heparin-binding, epidermal growth factor [15]. The vasoconstriction that was induced by the stimulation of the adrenoceptor (alpha-1B) with phenylephrine in the perfused artery was attenuated by blocking EGFR, the heparin-binding epidermal growth factor, and MMP [16]. Similar to previous reports, the contraction induced by the alpha-2 adrenoceptor agonist dexmedetomidine was attenuated by the EGFR inhibitor AG1478 and the MMP inhibitor GM6001 (Figure 1A and Figure 2D) [15,16]. Although dexmedetomidine is a highly selective alpha-2 adrenoceptor agonist (alpha-2/alpha-1 ratio = 1620) compared with the alpha-2 adrenoceptor agonist clonidine (alpha-2/alpha-1 ratio = 220), dexmedetomidine can also activate the alpha-1 adrenoceptor [17]. In addition, phenylephrine-induced contraction is partially mediated by the transactivation of EGFR [18]. EGFR mediates phenylephrine-induced extracellular-signal-regulated kinase phosphorylation and increases calcium response in vascular smooth muscle [19]. Thus, we examined the effect of the alpha-1 adrenoceptor inhibitor prazosin on dexmedetomidine-induced contraction. Dexmedetomidine-induced contraction was attenuated by prazosin (Figure 1B). As such, dexmedetomidine-induced contraction was attenuated by AG1478 in endothelium-denuded aorta pretreated with prazosin (Figure 1C). In addition, the alpha-2 adrenoceptor inhibitor rauwolscine inhibited dexmedetomidine-induced contraction (Figure 1D). Taken together, dexmedetomidine-induced contraction appears to be mediated mainly via the alpha-2 adrenoceptor and partially by the alpha-1 adrenoceptor. The EGFR tyrosine kinase inhibitor AG1478 could inhibit the alpha-1 adrenoceptor agonist phenylephrine-induced contraction without competitive inhibition [18]. Similar to previous findings, as AG1478 (10^−5^ M) inhibited dexmedetomidine-induced maximal contraction (Figure 1A), the AG1478-mediated inhibition of dexmedetomidine-induced contraction was most likely associated with the non-competitive inhibition of the alpha-2 adrenoceptor [18,20]. As agonist and antagonist binding sites are different in non-competitive inhibition, agonists do not displace antagonists [20]. Moreover, in terms of the magnitude of inhibition, a high concentration of AG1478 (3 × 10^−6^ M) inhibited the dexmedetomidine-induced contraction of endothelium-denuded rat aortas pretreated with rauwolscine (10^−6^ M) to a greater extent than a low concentration of AG1478 (10^−6^ M) (Figure 2A). This may be associated with allosteric inhibition, which could contribute to the enhanced inhibition of dexmedetomidine-induced contraction upon the combined treatment with rauwolscine and AG1478 compared with the alpha-2 adrenoceptor inhibitor rauwolscine alone. Thus, further studies are needed to examine allosteric inhibition, structural links, and ligand comparisons for both receptors (the alpha-2 adrenoceptor and EGFR) to elucidate the detailed mechanism underlying this phenomenon. In addition, the Src kinase inhibitors PP1 and PP2 and the MMP inhibitor GM6001 inhibited dexmedetomidine-induced contraction (Figure 2), suggesting that dexmedetomidine-induced contraction is mediated by cellular signal proteins involving EGFR, Src kinase, and MMP. Taken together with previous reports, the dexmedetomidine-induced transactivation of EGFR, which contributes to vasoconstriction, seems to be mediated by the alpha-2 adrenoceptor, Src kinase, and MMP [9,13,15,16]. Based on the above-mentioned results of the tension study, we investigated the underlying cellular signaling pathway that is associated with the dexmedetomidine-induced EGFR transactivation in rat aortic vascular smooth muscle. MMP mediates the transactivation of EGFR by activating G-protein-coupled receptors [21]. Angiotensin II induces the activation of an extracellular-signal-regulated kinase and the p38 mitogen-activated protein kinase, which is mediated by the metalloproteinase-dependent transactivation of EGFR in vascular smooth muscle [22]. The stimulation of the alpha-2B adrenoceptor by alpha-2 agonists in renal tubular cell lines activates MMP, the heparin-binding epidermal growth factor, and the transactivation of EGFR, leading to mitogen-activated protein kinase phosphorylation [13]. Consistent with the tension study and similar to previous reports, the alpha-2 adrenoceptor inhibitor rauwolscine, in addition to AG1478, PP1, PP2, and the MMP inhibitor GM6001, attenuated dexmedetomidine-induced EGFR phosphorylation (at Tyr1068; Figure 3), suggesting that this phosphorylation is mediated by the pathway involving the alpha-2B adrenoceptor, Src kinase, and MMP in vascular smooth muscle [13,23]. Further studies are needed to examine the mechanisms underlying the shedding of EGFR-specific ligands.

Dexmedetomidine-induced contraction involves JNK phosphorylation [11]. EGFR transactivation occurs via G-protein-coupled receptor agonists, which are associated with downstream signaling pathways, such as JNK, phosphoinositide 3-kinase, and extracellular-signal-regulated kinase [9]. Rauwolscine, PP1, PP2, GM6001, and AG1478 inhibited dexmedetomidine-induced JNK phosphorylation (Figure 4), suggesting that JNK phosphorylation occurs via a cell signaling pathway that is distal to the EGFR transactivation of dexmedetomidine mediated by the alpha-2 adrenoceptor, Src kinase, and MMP. We confirmed the relationship between EGFR transactivation and JNK phosphorylation, which are involved in dexmedetomidine-induced contraction, using vascular smooth muscle cells that were transfected with EGFR siRNA. Dexmedetomidine treatment increased JNK phosphorylation in vascular smooth muscle cells transfected with scrambled siRNA, whereas dexmedetomidine-induced JNK phosphorylation was attenuated in vascular smooth muscle cells transfected with EGFR siRNA (Figure 5). This result indicated that the dexmedetomidine-induced EGFR transactivation could induce JNK phosphorylation.

Dexmedetomidine-induced contraction is mainly dependent on the calcium influx that occurs via voltage-gated calcium channels [12]. In addition, epidermal growth factor induces vasoconstriction and increases the cytosolic free calcium concentration [10]. AG1478 attenuated the simultaneous [Ca^2+^]_i_ and contraction that was induced by the dexmedetomidine treatment (Figure 6A,B). Furthermore, AG1478 did not significantly alter the slope of the [Ca^2+^]_i_–tension relationship that was induced by dexmedetomidine (Figure 7A), suggesting that dexmedetomidine-induced contraction via EGFR transactivation is mediated by increased calcium levels, which mediates calcium-dependent contraction. ATP-sensitive potassium channels have been reported to be involved in epidermal growth factor-induced lung epithelial cell repair [24]. However, when treatments were combined with the ATP-sensitive potassium channel inhibitor glibenclamide and AG1478, it still did not significantly alter the slope of the calcium concentration–tension relationship that was induced by dexmedetomidine treatment compared with AG1478 alone (Figure 7B). This suggests that dexmedetomidine-induced [Ca^2+^]_i_ increase and contraction via EGFR transactivation are not modulated by ATP-sensitive potassium channels.

The dexmedetomidine-induced contraction mediated by the transactivation of EGFR partially contributes to increased blood pressure due to the administration of a high dose of dexmedetomidine [6,7,25]. However, this study has some limitations. First, small resistance arterioles are mainly involved in the peripheral vascular resistance associated with blood pressure, whereas we used the aorta in our study, which is a conduit vessel [26]. Second, this was an in vitro study using isometric tension measurements in isolated rat aortas. Microperfusion arteriography is a more reasonable method for measuring changes in artery diameter [16].

## 4. Materials and Methods

The experimental protocols used in this study (GNU-211215-R0103, 2 January 2022) were approved by the Institutional Animal Care and Use Committee at Gyeongsang National University. All experimental protocols were performed according to the guidelines of care and use of laboratory animals, prepared by the National Institute of Health.

### 4.1. Preparation of Isolated Rat Aortas and Isometric Tension Measurement

Male Sprague Dawley rats (body weight: 220–300 g, Koatecch, Pyeongtaek, Gyeonggi-do, Korea) were anesthetized with 100% carbon dioxide supplied into a small hole in a rat cage. The preparation of isolated rat aortas for isometric tension measurements was performed as previously described [27]. The thorax of the rats was exposed by sternotomy, and the descending thoracic aorta was extracted from the thorax. The thoracic aorta was immersed in Krebs solution containing sodium chloride (118 mM), sodium bicarbonate (25 mM), glucose (11 mM), potassium chloride (4.7 mM), calcium chloride (2.4 mM), magnesium sulfate (1.2 mM), and monopotassium phosphate (1.2 mM). The connective tissues and fat that were immersed in the Krebs solution were removed using scissors under a microscope (Carl Zeiss, Oberkochen, Germany). The isolated rat aorta was cut into 2.5 mm segments. The endothelium of the isolated rat aorta was removed by rolling the aorta using two 25-gauge needles inserted into the lumen of the rat aorta. The isolated thoracic aorta was suspended in a Grass isometric transducer (FT-03, Grass Instrument, Quincy, MA, USA) and attached to an organ bath, maintained at 37 °C. A baseline resting tension of 24.5 mN was maintained for 120 min to reach equilibrium. The Krebs solution was exchanged with fresh Krebs solution every 30 min during the equilibrium phase. The pH of the Krebs solution was maintained at 7.4 by supplying the Krebs solution with gas containing 95% oxygen and 5% carbon dioxide. Endothelial denudation of isolated rat aorta was confirmed using the following method: the endothelium-denuded aorta was precontracted with 10^−8^ M phenylephrine, and then 10^−5^ M acetylcholine was added to the Krebs solution containing the endothelium-denuded rat aorta precontracted with phenylephrine. The aortas showing less than 20% acetylcholine-induced relaxation following phenylephrine-induced contraction were regarded as endothelium-denuded aorta. The aorta showing acetylcholine-induced relaxation was washed several times with fresh Krebs solution to restore the baseline resting tension. After baseline resting tension was obtained, contraction was induced by adding isotonic 60 mM KCl into the organ bath. The measurement was obtained and used as a reference value to express the magnitude of dexmedetomidine-induced contraction. Then, the aorta was washed with fresh Krebs solution several times so that baseline resting tension was recovered, and the experimental protocols were then performed.

First, the effects of the EGFR tyrosine kinase inhibitor AG1478 on the dexmedetomidine-induced contraction of the isolated endothelium-denuded rat aortas were examined with or without the alpha-1 adrenoceptor inhibitor prazosin. Some of the endothelium-denuded rat aortas were pretreated with prazosin (10^−9^ M) for 10 min, followed by treatment with 10^−6^, 3 × 10^−6^, or 10^−5^ M AG1478 for 20 min. Then, 10^−9^ to 10^−6^ M dexmedetomidine was cumulatively added to the organ bath to generate dexmedetomidine concentration–response curves. In addition, the effect of the alpha-1 adrenoceptor inhibitor prazosin on the dexmedetomidine-induced contraction was examined. Some of the endothelium-denuded rat aortas were pretreated with prazosin (10^−9^ M) for 30 min. Then, dexmedetomidine (10^−9^ to 10^−6^ M) was cumulatively added to the organ bath to generate dexmedetomidine concentration–response curves in the presence or absence of prazosin. The effects of the alpha-2 adrenoceptor inhibitor rauwolscine alone and combined with AG1478 on dexmedetomidine-induced contraction in isolated endothelium-denuded rat aortas were examined. The isolated endothelium-denuded aortas were pretreated with rauwolscine (10^−6^ M) alone for 35 min or rauwolscine (10^−6^ M) for 20 min followed by AG1478 (10^−6^ or 3 × 10^−6^) for 15 min. Next, dexmedetomidine (10^−9^ to 10^−6^ M) was cumulatively added into the organ bath to generate concentration–response curves.

Second, the effects of the Src kinase inhibitors PP1 and PP2 and the MMP inhibitor GM6001 on dexmedetomidine-induced contraction in the isolated endothelium-denuded rat aortas were examined. The endothelium-denuded rat aortas were pretreated with PP1 (5 × 10^−6^, or 10^−5^ M), PP2 (3 × 10^−6^, or 10^−5^ M), and GM6001 (10^−5^ or 3 × 10^−5^ M) for 20 min. Then, dexmedetomidine (10^−9^ to 10^−6^ M) was cumulatively added to the organ bath to generate dexmedetomidine concentration–response curves in the presence or absence of PP1, PP2, and GM6001. In addition, the effects of DMSO (0.1%) and ethanol (0.1%) on dexmedetomidine (10^−9^ to 10^−6^ M)-induced contraction were examined in endothelium-denuded rat aortas, as these solutions were used for the dissolution of AG1478, GM6001, PP1, and PP2.

### 4.2. Detection of EGFR and JNK Phosphorylation in Rat Aortic Smooth Muscle

The phosphorylation levels of EGFR (Tyr1068) and JNK were assessed in the endothelium-denuded rat aortas using Western blotting with specific antibodies, as previously described [23,27,28]. Isolated endothelium-denuded rat aortic strips were treated with various inhibitors (10^−5^ M AG1478, 3 × 10^−5^ M GM6001, 10^−6^ M rauwolscine, or 10^−5^ M PP1 and PP2) for 30 min followed by dexmedetomidine (10^−6^ M) for 60 min, using inhibitors alone for 90 min, or with dexmedetomidine (10^−6^ M) alone for 60 min to detect EGFR phosphorylation. To detect JNK phosphorylation, the same process was followed with different time intervals. As such, various inhibitors were added for 30 min followed by the addition of dexmedetomidine for 10 min; alternatively, dexmedetomidine treatment alone was conducted for 10 min, or inhibitors alone were added for 40 min.

For the immunoblot analysis, aortic tissues were chopped and homogenized in radioimmunoprecipitation assay lysis buffer (Cell Signaling Technology, Beverly, MA, USA), containing a protease inhibitor cocktail (Thermo Fisher Scientific, Rockfield, IL, USA) and phosphatase inhibitor cocktail (Thermo Fisher Scientific), to obtain tissue lysates. The aortic vascular smooth muscle lysates were then centrifuged at 20,000× *g* for 15 min at 4 °C to discard debris. The supernatant was collected, and the protein concentration was measured using a bicinchoninate protein assay reagent kit (Thermo Fisher Scientific). Proteins were denatured by boiling the lysates for 10 min, and then the proteins were separated by 8–10% sodium dodecyl sulfate-polyacrylamide gel electrophoresis. The separated proteins were transferred to a polyvinylidene difluoride membrane (Millipore, Bedford, MA, USA). After blocking with 5% bovine serum albumin or 5% non-fat dried milk in Tris-buffered saline with 0.5% Tween-20 (0.5% TBST) at room temperature (22–26 °C) for 1 h, the membranes were incubated with primary antibodies (anti-EGFR (Tyr1068; 1:500), anti-phospho-EGFR (1:3000), anti-JNK (1:1000), anti-phospho-JNK (1:1000), and anti-β-actin (1:10,000) antibody) at 4 °C overnight. After washing with TBST, the membranes were incubated with a horseradish peroxidase-conjugated anti-rabbit or anti-mouse IgG antibody diluted at 1:5000, for 1 h at room temperature. Immune complexes were detected using Westernbright^TM^ ECL Western blotting detection kit (Advansta, Menlo Park, CA, USA), and images were captured using the ChemiDoc^TM^ Touch Imaging System (Bio-Rad Laboratories Inc., Hercules, CA, USA). The densities of the bands were determined with Image Lab Software v.3.0 (Bio-Rad Laboratories, Inc., Hercules, CA, USA).

### 4.3. Cell Culture 

Vascular smooth muscle cells that were isolated from rat thoracic aortas (using the outlined process for Western blot lysate generation) were cultured in Dulbecco’s modified Eagle’s medium (Gibco, Life Technologies, Grand Island, NY, USA) supplemented with 10% fetal bovine serum (Gibco), 100 units/mL penicillin, and 100 μg/mL streptomycin (Gibco), as previously described [27]. The cells were incubated at 37 °C in a humidified atmosphere containing 5% CO_2_. Cells between passages 3 and 5 were starved in serum-free medium for 15 h before drug treatment.

### 4.4. Gene Silencing Experiments Using Small Interfering RNA (siRNA) 

Knockdown of EGFR expression was performed by employing RNA interference methods using siRNA constructs, as described previously [27,29]. The scrambled siRNA for non-target control and pre-designed EGFR siRNA were purchased from Bioneer (Daejeon, Korea). The siRNA sequences used in this study were as follows: scrambled siRNA sense sequence 5′-CCU ACG CCA AUU UCG U-3′; antisense sequence 5′-ACG AAA UUG GUG GCG UAG G-3′, and EGFR siRNA sense sequence 5′-GAG CAU UUG GCA CAG UGU A-3′, antisense sequence 5′-UAC ACU GUG CCA AAU GCU C-3′. Transfections in vascular smooth muscle cells were performed using Lipofectamine RNAiMax transfection reagent (Thermo Fisher Scientific, Carlsbad, CA, USA) according to the manufacturer’s instructions. Each siRNA construct was transfected in the culture medium supplemented with 10% serum at a final concentration of 50 nM. The effects of gene silencing were assessed by Western blot analysis.

### 4.5. Simultaneous Calcium Level ([Ca^2+^]_i_) and Tension Measurement Using Fura-2-Loaded Aortic Strips

[Ca^2+^]_i_ was measured using the fluorescent Ca^2+^ indicator, Fura-2, as previously described [27]. Rat aortic muscle strips were exposed to the acetoxymethyl ester of fura-2 (fura-2/AM, 5 μM) with 0.02% Cremophor EL for 5–6 h at room temperature. After Fura-2 loading, the muscle strip was washed with physiological salt solution at 37 °C for 20 min to remove uncleaved fura-2/AM, and it was held horizontally in a temperature-controlled 7 mL organ bath. The force-displacement transducer used to measure muscle contraction was connected to one end of the muscle strips. The muscle strips were illuminated alternatively (48 Hz) at two excitation wavelengths (340 and 380 nm). The intensity of the 500-nm fluorescence (F340/F380) was monitored using a fluorometer (CAF-100, Jasco, Tokyo, Japan). The F340/F380 ratio was measured as an indicator of [Ca^2+^]_i_. The absolute [Ca^2+^]_i_ was not measured because the dissociation constant of the fluorescence indicator of calcium in the cytosol may be different than from that obtained in vitro. Thus, the F340/F380 ratios obtained from the resting and 60 mM KCl-induced contraction were regarded as 0% and 100%, respectively. The F340/F380 ratio and isometric contraction were both recorded using powerLab/400 with a chart program (MLT050, AD Instruments, Colorado Springs, CO, USA). The muscle strips were placed under an initial 29.4 mN resting tension. All strips obtained from the same rats were used for each experimental protocol. Epidermal growth factor activates ATP-sensitive potassium channels in the lung epithelial repair process [24]. Thus, simultaneous [Ca^2+^]_i_ and tension measurements were performed by cumulative addition of dexmedetomidine (10^−9^ to 10^−6^ M) into an organ bath in the presence or absence of AG1478 alone or combined treatment with glibenclamide and AG1478. After aortic strips were treated with AG1478 alone for 10 min or 10^−5^ M glibenclamide for 10 min followed by 10^−5^ M AG1478 for 10 min, 10^−9^ to 10^−6^ M dexmedetomidine was cumulatively added to the organ bath to generate measurements of simultaneous [Ca^2+^]_i_ and tension, induced by dexmedetomidine.

### 4.6. Chemicals

All chemicals and drugs were of the highest purity and were commercially available. Dexmedetomidine was obtained from Orion Pharma (Turku, Finland). Phenylephrine, acetylcholine, AG1478, GM6001, PP1, PP2, glibenclamide, rauwolscine, and anti-β-actin antibody were all obtained from Sigma Aldrich (St. Louis, MO, USA). The anti-EGFR (Tyr1068), anti-JNK, and anti-phospho-JNK antibodies were obtained from Cell Signaling Technology. The anti-phospho-EGFR (Tyr1068) antibody was obtained from Abcam (Cambridge Science Park, Cambridge, UK). AG1478 was dissolved in ethanol at a final concentration of 0.1%. PP1, PP2, glibenclamide, and GM6001 were dissolved in DMSO (final concentration: 0.1%). The other drugs were dissolved in distilled water.

### 4.7. Statistical Analysis

Data are shown as mean ± SD. Dexmedetomidine-induced contraction is expressed as a percentage of isotonic 60 mM KCl-induced contraction. The magnitude of simultaneous [Ca^2+^]_i_ and vasoconstriction induced by dexmedetomidine is expressed as a percentage of [Ca^2+^]_i_ and vasoconstriction induced by 60 mM KCl. The effects of various inhibitors on dexmedetomidine-induced contraction, and simultaneous [Ca^2+^]_i_ and vasoconstriction induced by dexmedetomidine, were analyzed using the linear mixed effect model (Stata version 14.2, Stata Crop LP, Lakeway Drive, College Station, TX, USA) [30]. The effects of various inhibitors on EGFR and JNK phosphorylation induced by dexmedetomidine, and the slope of simultaneous [Ca^2+^]_i_ and vasoconstriction induced by dexmedetomidine, were analyzed using a one-way analysis of variance followed by Bonferroni’s test (Prism 5.0, GraphPad Software, Inc., San Diego, CA, USA). Statistical significance was set at *p* < 0.05.

## 5. Conclusions

Taken together, these results suggest that the transactivation of EGFR by dexmedetomidine, which is associated with contraction of the vascular smooth muscle, is mediated by alpha-2 adrenoceptor, Src kinase, and MMP. Dexmedetomidine-induced contraction, which is mediated partially by the transactivation of EGFR, also involved JNK phosphorylation and increased calcium levels. This dexmedetomidine-induced transactivation of EGFR may contribute to increased blood pressure induced by a high dose of dexmedetomidine. 

## Data Availability

The data presented in this study are available on reasonable request from the corresponding author.

## Supplementary Materials

The following supporting information can be downloaded at: https://www.mdpi.com/article/10.3390/ijms23084320/s1.
