# Supplementary material for: Dexmedetomidine-Induced Aortic Contraction Involves Transactivation of the Epidermal Growth Factor Receptor in Rats
Source: Int J Mol Sci. 2022 Apr 13;23(8):4320. doi: 10.3390/ijms23084320 (PMC9024600; doi:10.3390/ijms23084320)
Supplement: Supplementary file 1 [file ijms-23-04320-s001.zip › ijms-1657479-supplementary.pdf]

Supplementary material

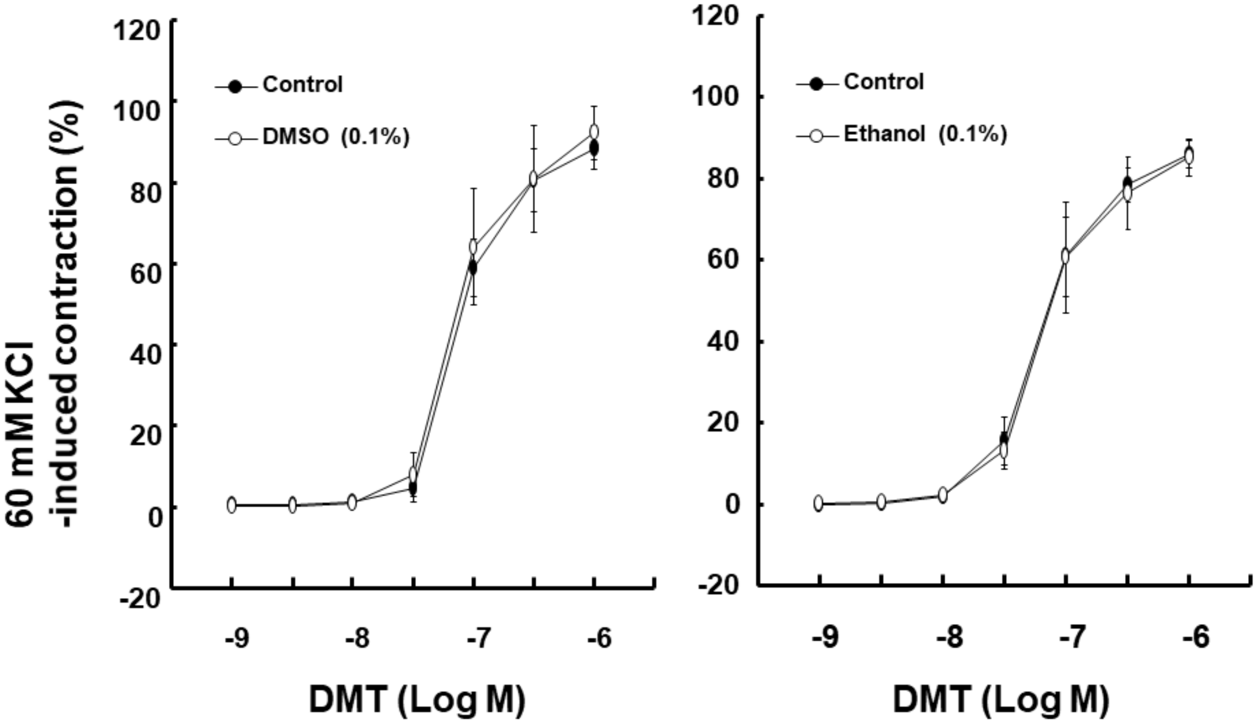

Figure S1. Effect of dimethyl sulfoxide (DMSO; N = 5), and ethanol (N = 6) on the dexmedetomidine (DMT)-induced contraction in isolated endothelium-denuded rat aortas. Data are shown as the mean ± SD and expressed as the percentage of isotonic 60 mM KCl-induced contraction. Control indicates no treatment. N indicates the number of isolated rat aorta.
